# Prevalence of vitamin D insufficiency in early pregnancies– a Singapore study

**DOI:** 10.1371/journal.pone.0300063

**Published:** 2024-04-11

**Authors:** Chua Zu’Er Amelia, Chan Hiu Gwan, Tan Shu Qi, John Tee Chee Seng

**Affiliations:** 1 Department of Obstetrics and Gynaecology, KK Women’s and Children’s Hospital, Singapore, Singapore; 2 Obstetrics and Gynaecology Academic Clinical Programme (OBGYN ACP), SingHealth Services, Singapore, Singapore; 3 Department of Paediatrics, Endocrinology Service, KK Women’s and Children’s Hospital, Singapore, Singapore; Nanjing Medical University, CHINA

## Abstract

Vitamin D plays an essential role in bone and mineral metabolism. There is increased interest in understanding prevalence of Vitamin D deficiency in pregnancy as many studies report association of low vitamin D levels with obstetric complications and neonatal sequelae. There is a paucity of studies in Singapore evaluating levels of vitamin D levels during the first trimester of pregnancies. We aim to study the prevalence of vitamin D insufficiency in this population. Our study assessed vitamin D levels in these women. Vitamin D (Plasma 25(OH)D concentration) levels in multiracial women during the first trimester were collected via venepuncture at their booking antenatal visit. They were stratified into sufficient ≥30ng/ml, insufficient ≥20ng/ml and <30ng/ml, moderately deficient ≥10ng/ml and <20ng/ml and severely deficient <10ng/ml. 93 women were included in this study. Only 2.2% of our study population had sufficient vitamin D levels. In women who had insufficient levels, the heavier the weight, the more likely to be vitamin D deficient. Interestingly, we also note that the older the patient, the less likely they are to be deficient. In women with periconceptual multivitamin supplementation, the average vitamin D level for those with supplementation was 2.10ng/ml higher than those without. Majority of patients were recruited from a single study member’s patient pool who were mostly Chinese. Prevalence of Vitamin D deficiency in general obstetric patients with higher BMI and darker skinned patients may be even lower in Singapore. The high prevalence of Vitamin D insufficiency in our patients prove that it is a prominent problem in our population. We aim to implement screening of vitamin D levels as part of antenatal investigations in the first trimester and recommend supplementation as required. We also hope to evaluate the association of low vitamin D levels with obstetric or neonatal complications further understanding its implications.

## Introduction

**Fig 1:**
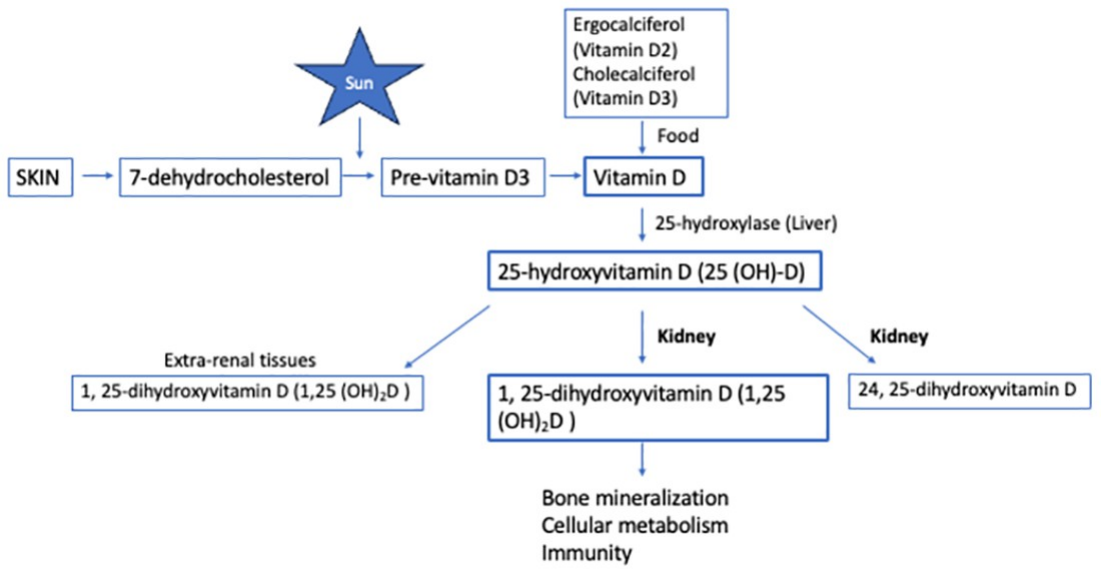
Vitamin D metabolism. This pathway shows the importance of 25-hydroxyvitamin D in bone mineralization, cellular metabolism and immunity.

Vitamin D plays an essential role in bone and mineral metabolism [1]. It is a fat soluble pro-hormone obtained mainly through action of sunlight on skin which converts 7-dehydrocholesterol to pre-vitamin D3. It is then metabolised to vitamin D3. Vitamin D can also be obtained from dietary sources such as milk and cheese. Dietary vitamin D exists as either ergocalciferol (Vitamin D2) or cholecalciferol (Vitamin D3) [2]. Liver 25-hydroxylase enzyme converts vitamin D2 and D3 to main circulating form of 25-hydroxyvitamin D (25 (OH)-D) Fig 1. 25 (OH)-D is used as biomarker for vitamin D status in view of its longer half-life. It is then converted by the kidneys and other tissues to active form of 1,25-dihydroxyvitamin-D (1,25 (OH)2D). Vitamin D is important in maintaining bone mineralization, cellular metabolism and immunity [3]. Nuclear receptors for 1,25 (OH)2D. are present in range of tissues including bone, intestine, kidney, lung, muscle and skin. Vitamin D helps to increase calcium absorption, inhibits PTH secretion and adaptive immunity. It has also been known to promote insulin secretion [4] and innate immunity.

Vitamin D deficiency has become an area of increased research interest as more evidence has revealed its relation to maternal and fetal complications during pregnancy. Some maternal complications include pre-eclampsia(PE) [5], gestational diabetes mellitus(GDM) [6] and intrauterine growth restriction(IUGR) [7]. Vitamin D is produced in the skin with sunlight exposure or artificial ultraviolet(UV) light. In darker-skinned people, sunlight is not as effective in producing Vitamin D as the skin pigment absorbs UV light [8]. Developed countries such as USA, the Netherlands and Korea have reported high prevalence of maternal hypovitaminosis D [9–11] in the first trimester especially in darker-skinned individuals with reduced sun exposure and higher body mass index (BMI). They have since established guidelines in prevention of vitamin D deficiency in their obstetric population [12]. The neighbouring country, Indonesia, also reports high prevalence of Vitamin D deficiency at 63% in a meta-analysis. [13] In Singapore, our studies on vitamin D have mainly looked at the general population and they have established that there is prevalence of hypovitaminosis D [14–16]. There is however a paucity of local data looking at pregnant patients although our patient profile do fall into high risk groups. It is postulated that hypovitaminosis D can occur in pregnancy secondary to inadequate sun exposure, fetal utilization and inadequate supplementation2. In one study by Baker et al [17] in the United States, it was reported that women with Vitamin D levels <20ng/ml had 4-fold increased risk of severe PE. Furthermore, women with early onset severe PE and small for gestational age (SGA) infants had significantly lower vitamin D levels than those with severe PE but non SGA infants [18]. This is further supported by a 2023 case-control study in Sweden establishing Vitamin D deficiency as a risk factor in early-onset pre-clampsia [19].

Maternal hypovitaminosis D is also associated with neonatal sequelae such as hypocalcemic convulsions [20], rickets [20] and asthma [21]. It has been shown to be a major cause of hypocalcemic seizures [22] in neonates and infants and plausible physiological mechanisms account for the relation between vitamin D status and immune development [23]. It is also associated with impaired growth and bone development in the fetus [24].

Our study assessed the vitamin D levels in our obstetric population in the first trimester of their pregnancies. Our aim was to establish the prevalence of vitamin D deficiency in early pregnancy in our local population as this is likely reflective of pre-conception Vitamin D levels. Also, This can then help us to determine the role of universal screening of vitamin D levels in our routine antenatal investigations. By supplementing those who are deficient, we can potentially reduce sequelae from maternal and fetal complications. The aim of our study was achieved as we established the high prevalence of Vitamin D insufficiency.

## Materials and methods

### Study design and setting

A prospective cohort study was conducted at the antenatal outpatient clinic of the KK Women’s and Children’s Hospital (KKH) in Singapore during the period of October 2020 to April 2021. This study was approved by the SingHealth Centralised Institutional Review Board (CIRB) (2020–2258).

### Patient selection

Our inclusion criteria were patients who were 21 years and older and in their first trimester (<15 weeks gestation) as determined by the crown rump length of their fetuses on ultrasound done in our centre’s Antenatal Monitoring Centre. Informed consent to participate in our study was sought by our team members for patients agreeable to participate in our study. Our exclusion criteria include patients with pre-existing diabetes mellitus, renal or cardiovascular diseases and people with anti-convulsant use. Maternal demographics such as gravida, parity, hours of sun exposure per day (in hours), consumption of peri-conception supplements, education and health factors were recorded via an investigator-led standardized questionnaire after informed consent had been taken by our study team members. Patients had their blood drawn at baseline in non-fasting state via venepuncture at their ultrasound visit and serum levels of 25-hydroxyvitamin D (25-OH Vit D) were recorded in our database. They were further stratified into sufficient ≥30ng/ml, insufficient ≥20ng/ml and <30ng/ml, moderately deficient ≥10ng/ml and <20ng/ml and severely deficient <10ng/ml [25].

### Measurements

Pregnancy and its duration were confirmed by our sonographers in the Antenatal Monitoring Clinic in KKH. Height and weight were measured during physical examination during their first antenatal consultation using standard procedure and body mass index (BMI) was calculated (kg/m^2^). A total of 5ml of blood was collected in a plain tube and standard assay of 25-OH Vit D was performed.

### Statistical analysis

Data were presented as frequency and percentage for categorical variables, and mean, standard deviation, median and Interquartile range for continuous variables. Pearson’s chi-square test was performed on categorical variables and analysis of variance was used for continuous variables. We classified level of 25-(OH)D lower than 20ng/ml as deficient while 20ng/ml or higher as not deficient, logistics regression was performed to examine the differences between the groups. Differences between level of 25-(OH)D and periconceptual multivitamin supplementation were examined using independent t-test. Sample size was validated by basing mean level of 25-(OH)D in van der Meer et al’s paper [10] to ensure that 95% of confidence interval estimate of proportion of 25-(OH)D deficiency in pregnant Singaporean women in their first trimester is within 10% of the true proportion. Spearman correlation coefficients were calculated to assess the association between variables.

Statistical analysis was performed using Statistical Package for the Social Sciences (SPSS) for Windows, version 19.0 (IBM Inc.). Statistical significance was set at 0.05.

## Results

In total, 93 pregnant women were recruited for the study. The mean (standard deviation, SD) age was 32.1 (3.69) years (age range 23–40 years). The average gestational age at recruitment was 12.2 weeks. The mean weight was 58.1kg with average BMI of 22.5. Within our population, the mean 25-OH Vit D levels were 20.0ng/ml (5.04). The median level was 20.1ng/ml. (Table 1).

**Table 1:** Demographics of pregnant women included in study.

	Age	Gestational age	Height (m)	Weight (kg)	BMI	Vitamin D levels (ng/ml)
Mean	32.1	12.2	1.61	58.1	22.5	20.0
Median	32.0	12.4	1.61	57.0	21.6	20.1
Standard Deviation	3.69	1.33	0.058	11.9	4.11	5.04
Minimum	23	9.00	1.47	36.0	15.2	7.00
Maximum	40	15.1	1.76	118.0	40.8	33.2

**Fig 2:**
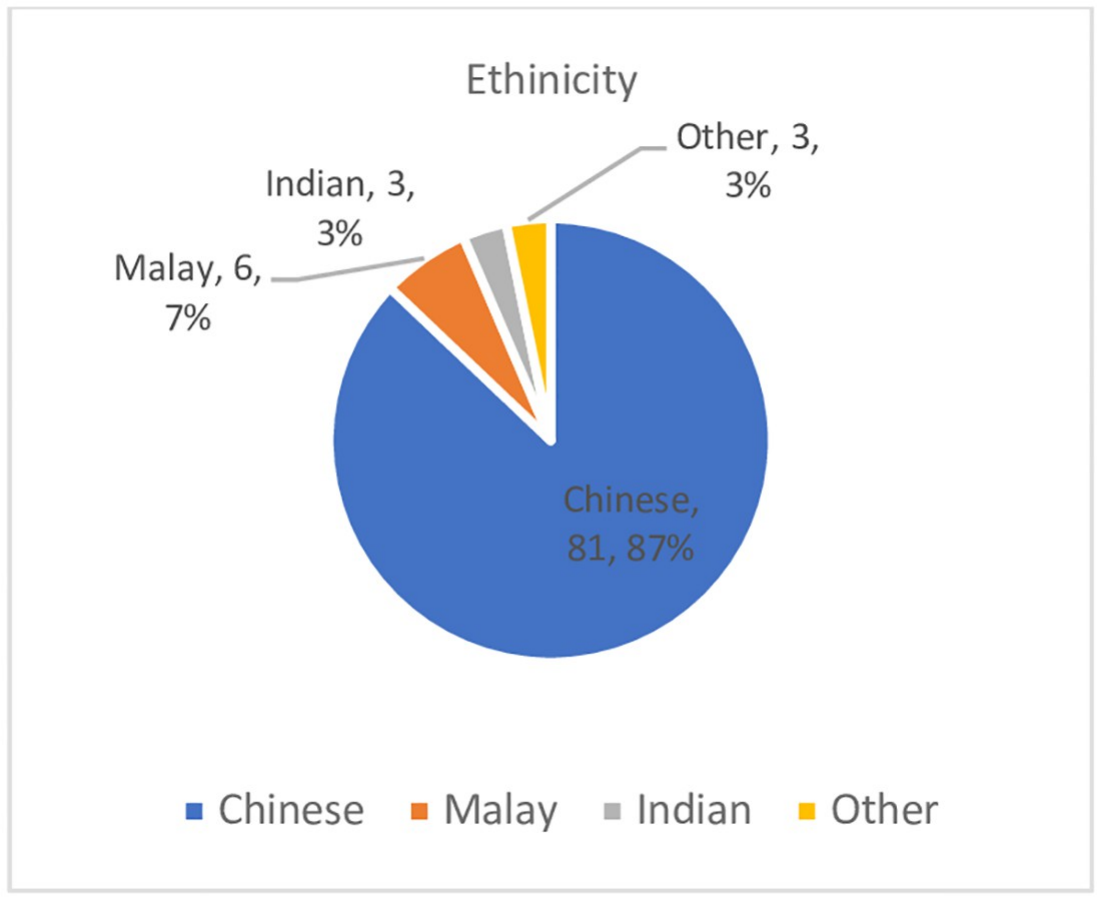
Ethnicity of study population. Majority (87%) of our study population were Chinese.

87% of the recruited patients were Chinese, 7% were Malay, 3% Indians and 3% of other races. Fig 2.

The characteristics of the study population are summarized and stratified based on Vitamin D levels (Table 2). Only 2.2% of our study population had sufficient vitamin D levels. Of the rest of the participants, 49.5% were vitamin D insufficient, 46.2% were moderately deficient and 2.2% were severely deficient. All women who were severely deficient were Chinese. None of the Indians or Malays included in this study had sufficient Vitamin D levels.

**Table 2:** Characteristics of pregnant women included in study stratified based on Vitamin D levels.

Category	Severely Deficient	Moderately Deficient	Insufficient	Sufficient
Vitamin D level (ng/mL)	< 10	≥10 and < 20	≥20 and < 30	≥30
No of subjects	N = 2	N = 43	N = 46	N = 2
**Continuous variables**	Mean, +/-SD, Median, IQR (25%-75%)			
Age	30.5 ± 0.7 30.5, NA	31.3 ± 3.7 31.0 IQR (29.0–34.0)	32.8 ± 3.6 33.0 IQR (31.0–35.0)	32.5 ± 6.4 32.5, NA
Gestational Age (GA)	10.8 ± 0.3 10.8, NA	12.2 ± 1.2 12.4 IQR (11.3–13.0)	12.2 ± 1.4 12.4 IQR (10.7–13.6)	12.2 ± 1.4 12.4, NA
Weight (kg)	66.0 ± 1.4 66.0, NA	60.5 ± 14.5 58.0 IQR (51.9–68.0)	55.9 ± 8.7 55.0 IQR (49.0–63.0)	55.9 ± 8.7 55.0, NA
Height (m)	1.62 ± 0.06 1.62, NA	1.61 ± 0.05 1.63 IQR (1.58–1.64)	1.60 ± 0.06 1.60 IQR (1.56–1.65)	1.57 ± 0.05 1.57, NA
BMI	25.2 ± 1.2 25.2, NA	23.2 ± 4.9 21.8 IQR (20.2–26.8)	21.8 ± 3.2 21.4 IQR (19.3–24.1)	21.8 ± 3.2 21.4, NA
**Categorical variables**	(number), (row %)			
**Hours of Sun exposure/day (hours)**				
< 1 hour	0/47 (0.0%)	22/47 (46.8%)	24/47 (51.1%)	1/47 (2.1%)
≥ 1 hour and < 2 hours	1/27 (3.7%)	14/27 (51.9%)	11/27 (40.7%)	1/27 (3.7%)
≥ 2 hours	1/19 (5.3%)	7/19 (36.8%)	11/19 (57.9%)	0/19 (0.0%)
**Race**				
Chinese	2/81 (2.5%)	36/81 (44.4%)	41/81 (50.6%)	2/81 (2.5%)
Malay	0/6 (0.0%)	3/6 (50.0%)	3/6 (50.0%)	0/6 (0.0%)
Indian	0/3 (0.0%)	3/3 (100.0%)	0/3 (0.0%)	0/3 (0.0%)
Others	0/3 (0.0%)	1/3 (33.3%)	2/3 (66.7%)	0/3 (0.0%)
**Smoker**				
No	2/93 (2.2%)	43/93 (46.2%)	46/93 (49.5%)	2/93 (2.2%)
**Periconceptual multivitamin supplementation**				
No	2/54 (3.7%)	27/54 (50.0%)	25/54 (46.3%)	0/54 (0.0%)
Yes	0/39 (0.0%)	16/39 (41.0%)	21/39 (53.8%)	2/39 (5.1%)
**Gravida**				
0–1	2/34 (5.9%)	13/34 (38.2%)	19/34 (55.9%)	0/34 (0.0%)
2	0/45 (0.0%)	24/45 (53.3%)	19/45 (42.2%)	2/45 (4.4%)
3–4	0/14 (0.0%)	6/14 (42.9%)	8/14 (57.1%)	0/14 (0.0%)
**Parity**				
0	1/38 (2.6%)	15/38 (39.5%)	22/38 (57.9%)	0/38 (0.0%)
1–2	1/55 (1.8%)	28/55 (50.9%)	24/55 (43.6%)	2/55 (3.6%)
**Education**				
Secondary/JC/Polytechnic/vocation/ITE	0/25 (0.0%)	12/25 (48.0%)	12/25 (48.0%)	1/25 (4.0%)
University & above	2/68 (2.9%)	31/68 (45.6%)	34/68 (50.0%)	1/68 (1.5%)

None of our participants were smokers. Of all women included, 41.9% reported taking periconceptual multivitamin supplementation. All reported that their multivitamins included vitamin D. No participants taking supplementation had severely deficient vitamin D levels. 36.6% of them were primigravida. The two participants who were severely vitamin D deficient were primigravida but the majority of participants (61.2%) who were vitamin D insufficient or deficient were multigravida. 73.1% of our patients received tertiary level of education. (Table 2).

Our research also showed that there is a negative correlation between Vitamin D levels and BMI which is statistically significant (Table 3). This corresponds to Walsh et al’s paper which found low vitamin D levels across age, ethnicity and geography [26] when correlated to BMI. Interestingly, there is also a positive relation between Vitamin D level and age which is statistically significant. However, when we categorize patients into two groups where there are Vitamin D deficient or not, age is not statistically significant in the logistic regression model (0.89, (0.79, 1.00); p = 0.052) (Table 4). Also for multivariable analysis, age is not statistically significant to determine Vitamin D deficient when weight is also taken into consideration.

**Table 3:** Correlation between variables.

	Vitamin D Levels (ng/mL)	BMI	Age	Weight (kg)	Education	Hours of Sun Exposure
Vitamin D Levels (ng/mL)	1	-0.287 p = 0.005	0.207 p = 0.046	-0.282 p = 0.006	0.016 p = 0.883	-0.034 p = 0.747
BMI	-0.287 p = 0.005	1	0.077 p = 0.460	0.907 p < 0.0005	0.002 p = 0.985	0.273 p = 0.008
Age	0.207 p = 0.046	0.077 p = 0.460	1	0.024 p = 0.821	0.131 p = 0.209	-0.002 p = 0.987
Weight (kg)	-0.282 p = 0.006	0.907 p < 0.0005	0.024 p = 0.821	1	0.105 p = 0.315	0.290 p = 0.005
Education	0.016 p = 0.883	0.002 p = 0.985	0.032 p = 0.761	0.105 p = 0.315	1	0.164 p = 0.116
Hours of Sun Exposure	-0.034 p = 0.747	0.273 p = 0.008	-0.002 p = 0.987	0.290 p = 0.005	0.164 p = 0.116	1

**Table 4:** Association of Vitamin D levels and characteristic of pregnant women.

Dependent variable: Vitamin D Level (Deficient, < 20ng/mL)	Univariate	Multivariable
	Odds ratio (95% CI) p value	Odds ratio (95% CI) p value
Age	0.89 (0.79, 1.01) p = 0.052	0.89 (0.79, 1.00) p = 0.050
Weight (every 5 kg increase)	1.23 (1.01, 1.50) p = 0.043	1.24 (1.01, 1.53) p = 0.042
Height (every 10 cm increase)	1.54 (0.75, 3.18) p = 0.241	
BMI	1.10 (0.99, 1.23) p = 0.079	
Gestational Age	0.90 (0.66, 1.23) p = 0.510	
Education		
Secondary/JC/Polytechnic/vocation/ITE	Reference	
University & above	1.02 (0.41, 2.56) p = 0.964	
Gravida		
0–1	Reference	
2	1.45 (0.59, 3.54) p = 0.418	
3–4	0.95 (0.27, 3.34) p = 0.936	
Parity		
0	Reference	
1–2	1.53 (0.67, 3.53) p = 0.315	
Smoker	100% non-smoker	
Periconceptual Multivitamin Supplementation		
No	Reference	
Yes	0.60 (0.26, 1.38) p = 0.229	
Hours of Sun Exposure/day (hours)		
< 1 hour	Reference	
≥ 1 hour	1.14 (0.51, 2.56) p = 0.758	

In analyses including interaction between variables (age, weight, BMI, gestational age, education, gravida/parity, supplementation, hours of sun exposure) for suboptimal Vitamin D level, all were non-significant except for weight. We observed for every 5kg increase of weight, it is 23% more likely that the individual has vitamin D deficiency (odds ratio 1.23, 95%CI (1.01, 1.50); p = 0.043). (Table 4). It remains statistically significant when adjusting for age (1.24 (1.01, 1.53); p = 0.042).

The average vitamin D level of those who have periconceptual multivitamin supplementation is 2.10 ng/mL higher than those who does not have. It is statistically significant with p value = 0.047. 2 sample independent t test was conducted to assess the relationship between vitamin D level and periconceptual multivitamin supplementation.

Based on our analysis, participants who have < 1 hour of Sun Exposure per day is 2 times more likely that they are on Vitamin D Supplementation as compared to those who have ≥ 1 hour of Sun Exposure per day. (Table 5).

**Table 5:** Association of periconceptual multivitamin supplements and pregnant women.

Dependent variable: Periconceptual Multivitamin Supplementation (Yes—1, No—0)	Univariate
	Odds ratio (95% CI) p value
Age	0.99 (0.88, 1.11) p = 0.840
Weight (every 5 kg increase)	0.97 (0.82, 1.16) p = 0.763
Height (every 10 cm increase)	1.10 (0.54, 2.25) p = 0.801
BMI	0.97 (0.88, 1.08) p = 0.605
Gestational Age	1.29 (0.94, 1.78) p = 0.121
Education ß Secondary/JC/Polytechnic/vocation/ITE	Reference
University & above	0.89 (0.35, 2.25) p = 0.807
Gravida	
0–1	Reference
2	1.29 (0.52, 3.20) p = 0.580
3–4	1.21 (0.34, 4.29) p = 0.766
Parity	
0	Reference
1–2	1.19 (0.51, 2.75) p = 0.689
Smoker	100% non-smoker
Vitamin D Level	
≥ 20ng/mL	1.67 (0.73, 3.83) p = 0.229
< 20ng/mL	Reference
Hours of Sun Exposure/day (hours)	
< 1 hour	2.60 (1.11, 6.08) p = 0.028
≥ 1 hour	Reference

## Discussion

Our results, using the current cut-offs show a high prevalence of hypovitaminosis D in our population. There may be several reasons explaining this result i.e. approximately half of Singaporean workers hold indoor jobs in the professional, managerial, executive and technical (PMET) sectors limiting exposure to sunlight [27], a multi-ethnic population including Malays who traditionally are dressed for modesty leaving only face and hands exposed and a cultural notion amongst Asian Chinese that being fairer is beautiful [28].

In our study, we also note that all Indians and Malays recruited were Vitamin D deficient. This can be explained by increased skin pigmentation seen amongst these ethnicities. With more melanin, it’s been shown to absorb more ultraviolet radiation causing less cutaneous production of Vitamin D [29]. These ethnicities made up 7% and 3% of our study population. In actuality, our population consists of 75.7% Chinese, 15.2% Malay, 7.5% Indian and 1.6% of other ethnicities [30]. Our study is thus limited by the small sample size of Malays and Indians and may have overestimated the actual prevalence of vitamin D deficiency in them. However, studies done specifically amongst the Chinese in Beijing, China by Song et al [31] and in India by Sofi et al [32] also have shown that there were also high prevalence of hypovitaminosis D at 96.8% and 88% respectively. Thus, it is important to consider screening these high-risk populations who are darker skinned for vitamin D deficiency.

With our study, we have further supported other papers that have shown correlation between increased BMI with low vitamin D levels [33–35]. This can be understood to be due to reduced bioavailability of vitamin D from either cutaneous or dietary sources because of its deposition in fatty tissues [36]. The increased surface area and mass also contributes to lower vitamin D levels through volumetric dilution [37]. Hence, for patients identified with high BMI, screening should be recommended and supplementation should be considered. Dosage for these patients is likely to be higher than the general population as well to account for its reduced bioavailability. Drincic and colleagues [37]concluded that Vitamin D dosing should be weight-adjusted and stipulated that an intake of 1.25–2 mcg/kg/day (70–80 IU/kg/day) is expected to produce serum 1,25 (OH)_2_D in the 30–40 ng/ml range.

Aging is known to affect Vitamin D metabolism and calcium formation through the following: reduced calcium absorption, intestinal resistance of calcium absorption to circulating 1,25 (OH)_2_D, reduced concentration of intestinal Vitamin D receptor, reduced renal production of 1,25 (OH)_2_D, reduced skin production of Vitamin D and substrate deficiency of Vitamin D [38]. However, we noted that older participants in our study tend to be less deficient in Vitamin D. Although age does affect vitamin D metabolism, it usually occurs when individuals are older and typically beyond 65 years of age [39, 40] which can explain why older patients in the study were not more deficient in Vitamin D levels. Also, using a logistic regression model, age was not statistically significant (Table 4). This means that is not considered a good predictor to predict whether someone is Vitamin D deficient or not. Hence, age in pregnant patients is not important when we want to determine if an individual is vitamin D deficient or not.

Our hospital practises mainly along the guidelines set by the Royal College of Obstetrics and Gynaecology (RCOG) and the National Institute for Health and Care Excellence (NICE) from the United Kingdom. They currently recommend screening patients in high risk groups such as darker-skinned, limited exposure to sun and high BMI ≥30 [41, 42]. However, there is inapplicability of such guidelines to our local population who are predominantly Chinese with lighter skin. Locally, our Health Promotion Board advises 2.5mcg of vitamin D in our diet [43] but screening of Vitamin D deficiency has not been suggested in pregnant patients. The advice of 2.5mcg supplement is also well below the suggestion of 10mcg by RCOG and NICE. Our study suggests that standard supplementation of Vitamin D and screening of Vitamin D levels for at-risk groups should be advised in our local context.

Also, our study is unable to look at how seasonal trends affect vitamin D levels as our country is an equatorial country with no seasonal changes. To have such high prevalence of hypovitaminosis D in a sunny country is indeed an enigma. Another local study by Man et al done in 2016 also agrees with high prevalence of hypovitaminosis D in our general population at 75% [44].

## Strengths and limitations

Our study’s strength includes it being a pilot study in Singapore looking at Vitamin D levels amongst pregnant patients in their first trimesters. There is a lack of local studies looking at Vitamin D levels amongst women of reproductive age. However, there were also several limitations to our study. It was performed during the Coronavirus disease 2019 (COVID-19) restrictive period where group outdoor activities were restricted. There is the possibility that less outdoor activities could have contributed to lower Vitamin D levels in view of its physiology. Majority of our patients were also recruited from a single study member’s patient pool who were mostly Chinese. However, this may actually imply that the level of Vitamin D levels in our general obstetric patients with higher BMI and darker skinned colour may even be lower than what is established in our study. A longitudinal assessment of the study participants antenatally at each trimester and postpartum would also have helped to further look at associations between vitamin D levels and its reported maternal and fetal complications.

## Conclusion

Indeed, the prevalence of hypovitaminosis D was proven in our local cohort study. Moving forward, we hope to recommend Vitamin D supplementation in our population. We also hope to evaluate the association of low vitamin D levels with obstetric complications and aim to implement screening of vitamin D levels as part of antenatal investigations in the first trimester in our hospital.

## Supporting information

S1 Data De-identified data set. (XLSX)

## References

[1] Lips P, Van Schoor NM. The effect of vitamin D on bone and osteoporosis. Best practice & research Clinical endocrinology & metabolism. 2011;25(4):585–591. doi: 10.1016/j.beem.2011.05.002 21872800

[2] Holick MF. Vitamin D deficiency. New England journal of medicine. 2007;357(3):266–281. doi: 10.1056/NEJMra070553 17634462

[3] Baeke F, Takiishi T, Korf H, Gysemans C, Mathieu C. Vitamin D: modulator of the immune system. Current opinion in pharmacology. 2010;10(4):482–496. doi: 10.1016/j.coph.2010.04.001 20427238

[4] Tai K, Need AG, Horowitz M, Chapman IM. Vitamin D, glucose, insulin, and insulin sensitivity. Nutrition. 2008;24(3):279–285. doi: 10.1016/j.nut.2007.11.006 18187309

[5] Bodnar LM, Catov JM, Simhan HN, Holick MF, Powers RW, Roberts JM. Maternal vitamin D deficiency increases the risk of preeclampsia. The Journal of Clinical Endocrinology & Metabolism. 2007;92(9):3517–3522. doi: 10.1210/jc.2007-0718 17535985 PMC4288954

[6] Maghbooli Z, Hossein-nezhad A, Karimi F, Shafaei AR, Larijani B. Correlation between vitamin D3 deficiency and insulin resistance in pregnancy. Diabetes/metabolism research and reviews. 2008;24(1):27–32. doi: 10.1002/dmrr.737 17607661

[7] Scholl TO, Chen X. Vitamin D intake during pregnancy: association with maternal characteristics and infant birth weight. Early human development. 2009;85(4):231–234. doi: 10.1016/j.earlhumdev.2008.10.006 19008055

[8] Clemens T, Henderson S, Adams J, Holick M. Increased skin pigment reduces the capacity of skin to synthesise vitamin D3. The Lancet. 1982;319(8263):74–76. doi: 10.1016/S0140-6736(82)90214-8 6119494

[9] Bodnar LM, Simhan HN, Powers RW, Frank MP, Cooperstein E, Roberts JM. High prevalence of vitamin D insufficiency in black and white pregnant women residing in the northern United States and their neonates. The Journal of nutrition. 2007;137(2):447–452. doi: 10.1093/jn/137.2.447 17237325 PMC4288960

[10] van der Meer IM, Karamali NS, Boeke AJP, Lips P, Middelkoop BJ, Verhoeven I, et al. High prevalence of vitamin D deficiency in pregnant non-Western women in The Hague, Netherlands1, 2. The American journal of clinical nutrition. 2006;84(2):350–353. doi: 10.1093/ajcn/84.2.350 16895882

[11] Choi R, Kim S, Yoo H, Cho YY, Kim SW, Chung JH, et al. High prevalence of vitamin D deficiency in pregnant Korean women: the first trimester and the winter season as risk factors for vitamin D deficiency. Nutrients. 2015;7(5):3427–3448. 25970148 10.3390/nu7053427 PMC4446760

[12] of Obstetricians AC, Gynecologists, et al. Vitamin D: screening and supplementation during pregnancy. Committee Opinion No. 495. Obstet Gynecol. 2011;118:197–8. 21691184 10.1097/AOG.0b013e318227f06b

[13] Octavius GS, Daleni VA, Angeline G, Virliani C. A systematic review and meta-analysis of prevalence of vitamin D deficiency among Indonesian pregnant women: a public health emergency. AJOG Global Reports. 2023; p. 100189. doi: 10.1016/j.xagr.2023.100189 37234813 PMC10205541

[14] Bi X, Tey SL, Leong C, Quek R, Henry CJ. Prevalence of vitamin D deficiency in Singapore: its implications to cardiovascular risk factors. PLoS One. 2016;11(1):e0147616. doi: 10.1371/journal.pone.0147616 26799569 PMC4723156

[15] Ramason R, Selvaganapathi N, Ismail NHB, Wong WC, Rajamoney GN, Chong MS. Prevalence of vitamin d deficiency in patients with hip fracture seen in an orthogeriatric service in sunny singapore. Geriatric orthopaedic surgery & rehabilitation. 2014;5(2):82–86. doi: 10.1177/2151458514528952 25360336 PMC4212370

[16] Divakar U, Sathish T, Soljak M, Bajpai R, Dunleavy G, Visvalingam N, et al. Prevalence of vitamin D deficiency and its associated work-related factors among indoor workers in a multi-ethnic Southeast Asian country. International Journal of Environmental Research and Public Health. 2020;17(1):164. 10.3390/ijerph17010164 PMC6981433 31881679

[17] Baker AM, Haeri S, Camargo CA Jr, Espinola JA, Stuebe AM. A nested case-control study of midgestation vitamin D deficiency and risk of severe preeclampsia. The Journal of Clinical Endocrinology & Metabolism. 2010;95(11):5105–5109. doi: 10.1210/jc.2010-0996 20719829 PMC2968727

[18] Robinson CJ, Wagner CL, Hollis BW, Baatz JE, Johnson DD. Maternal vitamin D and fetal growth in early-onset severe preeclampsia. American journal of obstetrics and gynecology. 2011;204(6):556–e1. doi: 10.1016/j.ajog.2011.03.022 21507371 PMC3136573

[19] Malm G, Lindh CH, Hansson SR, Källén K, Malm J, Rylander L. Maternal serum vitamin D level in early pregnancy and risk for preeclampsia: A case-control study in Southern Sweden. Plos one. 2023;18(2):e0281234. doi: 10.1371/journal.pone.0281234 36749741 PMC9904465

[20] Camadoo L, Tibbott R, Isaza F. Maternal vitamin D deficiency associated with neonatal hypocalcaemic convulsions. Nutrition journal. 2007;6:1–2. doi: 10.1186/1475-2891-6-23 17880694 PMC2034574

[21] Thorsteinsdottir F, Cardoso I, Keller A, Stougaard M, Frederiksen P, Cohen AS, et al. Neonatal vitamin D status and risk of asthma in childhood: results from the D-Tect study. Nutrients. 2020;12(3):842. doi: 10.3390/nu12030842 32245170 PMC7146263

[22] Mehrotra P, Marwaha R, Aneja S, Seth A, Singla B, Ashraf G, et al. Hypovitaminosis d and hypocalcemic seizures in infancy. Indian pediatrics. 2010;47:581–586. doi: 10.1007/s13312-010-0131-1 20019397

[23] Walker VP, Zhang X, Rastegar I, Liu PT, Hollis BW, Adams JS, et al. Cord blood vitamin D status impacts innate immune responses. The Journal of Clinical Endocrinology & Metabolism. 2011;96(6):1835–1843. doi: 10.1210/jc.2010-1559 21470993 PMC3100757

[24] Morley R, Carlin JB, Pasco JA, Wark JD. Maternal 25-hydroxyvitamin D and parathyroid hormone concentrations and offspring birth size. The Journal of Clinical Endocrinology & Metabolism. 2006;91(3):906–912. doi: 10.1210/jc.2005-1479 16352684

[25] Grundmann M, von Versen-Höynck F. Vitamin D-roles in women’s reproductive health? Reproductive biology and endocrinology. 2011;9:1–12. doi: 10.1186/1477-7827-9-146 22047005 PMC3239848

[26] Walsh JS, Bowles S, Evans AL. Vitamin D in obesity. Current Opinion in Endocrinology, Diabetes and Obesity. 2017;24(6):389–394. doi: 10.1097/MED.0000000000000371 28915134

[27] Population N, (NPTD) TD. Population white paper:“A Sustainable Population for a Dynamic Singapore”; 2013.

[28] Kung AW, Lee KK. Knowledge of vitamin D and perceptions and attitudes toward sunlight among Chinese middle-aged and elderly women: a population survey in Hong Kong. BMC public health. 2006;6:1–7. doi: 10.1186/1471-2458-6-226 16956420 PMC1584409

[29] Jablonski NG, Chaplin G. The evolution of human skin coloration. Journal of human evolution. 2000;39(1):57–106. doi: 10.1006/jhev.2000.0403 10896812

[30] Prime Minister’s Office, Singapore. Population in Brief 2022: Key trends; 2022. https://www.population.gov.sg/media-centre/articles/permalink/.

[31] Song SJ, Zhou L, Si S, Liu J, Zhou J, Feng K, et al. The high prevalence of vitamin D deficiency and its related maternal factors in pregnant women in Beijing. PloS one. 2013;8(12):e85081. doi: 10.1371/journal.pone.0085081 24386450 PMC3873449

[32] Sofi NY, Jain M, Kapil U, Seenu V, Ramakrishnan L, Yadav CP, et al. Status of serum vitamin D and calcium levels in women of reproductive age in national capital territory of India. Indian Journal of Endocrinology and Metabolism. 2017;21(5):731–733. doi: 10.4103/ijem.IJEM_134_17 28989883 PMC5628545

[33] Parikh SJ, Edelman M, Uwaifo GI, Freedman RJ, Semega-Janneh M, Reynolds J, et al. The relationship between obesity and serum 1, 25-dihydroxy vitamin D concentrations in healthy adults. The Journal of Clinical Endocrinology & Metabolism. 2004;89(3):1196–1199. doi: 10.1210/jc.2003-031398 15001609

[34] Pereira-Santos M, Costa PdF, Assis Ad, Santos CdS, Santos Dd. Obesity and vitamin D deficiency: a systematic review and meta-analysis. Obesity reviews. 2015;16(4):341–349. doi: 10.1111/obr.12239 25688659

[35] Lagunova Z, Porojnicu AC, Lindberg F, Hexeberg S, Moan J. The dependency of vitamin D status on body mass index, gender, age and season. Anticancer research. 2009;29(9):3713–3720. 19667169

[36] Wortsman J, Matsuoka LY, Chen TC, Lu Z, Holick MF. Decreased bioavailability of vitamin D in obesity. The American journal of clinical nutrition. 2000;72(3):690–693. doi: 10.1093/ajcn/72.3.690 10966885

[37] Drincic AT, Armas LA, Van Diest EE, Heaney RP. Volumetric dilution, rather than sequestration best explains the low vitamin D status of obesity. Obesity. 2012;20(7):1444–1448. doi: 10.1038/oby.2011.404 22262154

[38] Gallagher JC. Vitamin D and aging. Endocrinology and Metabolism Clinics. 2013;42(2):319–332. doi: 10.1016/j.ecl.2013.02.004 23702404 PMC3782116

[39] Bullamore J, Wilkinson R, Gallagher J, Nordin B, Marshall D. Effect of age on calcium absorption. The lancet. 1970;296(7672):535–537. doi: 10.1016/S0140-6736(70)91344-9 4195202

[40] Ireland P, Fordtran JS, et al. Effect of dietary calcium and age on jejunal calcium absorption in humans studied by intestinal perfusion. The Journal of clinical investigation. 1973;52(11):2672–2681. doi: 10.1172/JCI107461 4748506 PMC302533

[41] Royal College of Obstetrician and Gynaecology, United Kingdom. Healthy eating and vitamin supplements in pregnancy; 2022. https://www.rcog.org.uk/media/lcfn54fw/healthy-eating-vitamin-supplements-pregnancy-large-print-patient-information.pdf.

[42] National Institute for Health and Care Excellance, United Kingdom. Vitamin D: supplement use in specific population groups; 2017. https://www.nice.org.uk/guidance/ph56.

[43] Health Promotion Board, Singapore. Recommended Dietary Allowances; 2023. https://www.healthhub.sg/live-healthy/recommended_dietary_allowances.

[44] Man REK, Li LJ, Cheng CY, Wong TY, Lamoureux E, Sabanayagam C. Prevalence and determinants of suboptimal vitamin D levels in a multiethnic Asian population. Nutrients. 2017;9(3):313. 28327512 10.3390/nu9030313 PMC5372976

